# Endonasal CNS Delivery System for Blood-Brain Barrier Impermeant Therapeutic Oligonucleotides Using Heterotopic Mucosal Engrafting

**DOI:** 10.3389/fphar.2021.660841

**Published:** 2021-04-19

**Authors:** Grishma Pawar, Neha N. Parayath, Aditya A. Sharma, Carlos Coito, Olga Khorkova, Jane Hsiao, William T. Curry, Mansoor M. Amiji, Benjamin S. Bleier

**Affiliations:** ^1^ Department of Pharmaceutical Sciences, School of Pharmacy, Northeastern University, Boston, MA, United States; ^2^ OPKO Health Inc., Miami, FL, United States; ^3^ Department of Neurosurgery, Harvard Medical School, Massachusetts General Hospital, Boston, MA, United States; ^4^ Department of Otolaryngology, Harvard Medical School, Massachusetts Eye and Ear, Boston, MA, United States

**Keywords:** central nervous system, blood-brain barrier, brain derived neurotrophic factor, natural antisense transcript, antisense oligonucelotides, liposomes

## Abstract

The most significant obstacle in the treatment of neurological disorders is the blood-brain barrier (BBB), which prevents 98% of all potential neuropharmaceuticals from reaching the central nervous system (CNS). Brain derived neurotrophic factor (BDNF) is one of the most intensely studied targets in Parkinson’s disease (PD) as it can reverse disease progression. BDNF AntagoNAT’s (ATs) are synthetic oligonucleotide-like compounds capable of upregulating endogenous BDNF expression. Despite the significant promise of BDNF AT therapies for PD, they cannot cross the blood-brain barrier (BBB). Our group has developed an innovative endonasal heterotopic mucosal grafting technique to provide a permanent method of permeabilizing the BBB. This method is based on established endoscopic surgical procedures currently used in routine clinical practice. Our overall goal for the study was to investigate the distribution and efficacy of BDNF AT’s using an extra-cranial graft model in naïve rats using the innovative heterotopic mucosal engrafting technique. BDNF AT cationic liposomes (ideal size range 200–250 nm) were developed and characterized to enhance the delivery to rat brain. Uptake, distribution and transfection efficiency of BDNF AntagoNAT’s in saline and liposomes were evaluated qualitatively (microscopy) and quantitatively (ELISA and AT hybridization assays) in RT4-D6P2T rat schwannoma cells and in naïve rats. *In vivo* therapeutic efficacy of BDNF AT’s encapsulated in liposomes was evaluated in a 6-OHDA toxin model of PD using western blot and tyrosine hydroxylase immunohistochemistry. Using complimentary *in vitro* and *in vivo* techniques, our results demonstrate that grafts are capable of delivering therapeutic levels of BDNF ATs in liposomes and saline formulation throughout the brain resulting in significant BDNF upregulation in key end target regions relevant to PD. BDNF AT liposomes resulted in a better distribution in rat brain as compared to saline control. The delivered BDNF AT’s encapsulated in liposomes also conferred a neuroprotective effect in a rat 6-OHDA model of PD. As a platform technique, these results further suggest that this approach may be utilized to deliver other BBB impermeant oligonucleotide-based therapeutics thereby opening the door to additional treatment options for CNS disease.

## Introduction

Neurological disorders account for up to 6.3% of the global disease burden (WHO, 2006). Although researchers have made considerable advances in our understanding of the pathogenesis and natural history of PD, the development of effective therapeutic options have failed to keep pace, in part because of the inability of most therapies to penetrate the blood-brain barrier (BBB).

Brain derived neurotrophic factor (BDNF) represents one of the most promising and intensely studied targets in neurodegenerative disease as it has been shown to delay or even reverse disease progression in PD (Ochs et al., 2000; Nagahara and Tuszynski, 2011; Ventriglia et al., 2013). However, exogenous delivery of recombinant neurotrophic factor-based therapies face several critical problems including dosing issues, off-target effects which have greatly hindered their clinical adoption (Krishna, 2016; Luz et al., 2016). Furthermore, recombinant protein manufacturing creates physiologic post-translational modifications which are then delivered to improper tissues and subcellular compartments (Quiroz et al., 2012). These concerns are germane to BDNF, where nine functional promoters, over 15 alternative transcripts, pre-protein processing and N-glycosylation, and a regulatory natural antisense transcript (NAT), all contribute to fine modulation of the biological activity (Mowla et al., 2001; Liu et al., 2005; Pruunsild et al., 2007).

NATs are a subclass of endogenous non-coding RNAs, which specifically inhibit the expression of many disease-related proteins through epigenetic mechanisms. NATs are only active in cells that constitutively express their respective target proteins (Katayama et al., 2005; Modarresi et al., 2012; Halley et al., 2014). Notably, most of the issues with post-translational modification and improper compartmentalization inherent to recombinant protein therapeutics can be avoided by designing therapies that modulate the activity of NATs using AntagoNATs (AT’s). AntagoNATs are oligonucleotide-based compounds complementary to a particular NAT sequence, and are highly specific to a single protein target (Magistri et al., 2012; Modarresi et al., 2012; Wahlestedt, 2013; Halley et al., 2014). Furthermore, AntagoNATs upregulate the endogenous protein in its natural subcellular environment where it is subjected to appropriate post-translational modifications and is fully accessible to endogenous feedback mechanisms thereby limiting toxicity. Unlike proteins which have to be produced in a biological host system, AntagoNATs are obtained by controlled chemical synthesis at a greatly reduced cost and do not contain potentially immunogenic carryover from the host cells. ATs solve the problems of recombinant BDNF therapies including off-target toxicity, immunogenicity, and improper post-translational modification. At the same time, they are capable of increasing target specificity, improving neuronal uptake, and providing appropriate subcellular compartmentalization of the expressed protein. BDNF NAT, expressed from the opposite strand of the BDNF genomic locus, is known to regulate BDNF expression and can therefore be targeted to increase endogenous BDNF expression for the treatment of neurodegenerative diseases. Our group has previously discovered multiple AntagoNAT sequences complementary to BDNF-NAT that increase BDNF expression with high potency and low toxicity using an established neurosphere assay (Modarresi et al., 2012). Despite the evident promise of BDNF AntagoNAT therapeutics in neurodegenerative disease, they are unable to cross the BBB thereby requiring delivery directly to the brain.

The major strategies that are currently being explored for brain delivery can be classified into invasive and non-invasive strategies (Dong, 2018). Invasive strategies like intraventricular, intrathecal and intracerebral injections involve physical disruption of the BBB thereby allowing drugs to enter the CSF and brain parenchyma (Lu et al., 2014). Intrathecal delivery of AntagoNAT-like compounds has been used in the clinic for several diseases including pediatric spinal muscular atrophy (Chiriboga et al., 2016; Finkel et al., 2017) and amyotrophic lateral sclerosis (Miller et al., 2013). While effective, intrathecal injections may be associated with significant morbidity including spinal headache, local back or radiating leg pain, meningitis, epidural abscess, subdural hematoma, and even death (Gaucher and Perez, 2002; Pandian et al., 2004). Similarly, other invasive injections involve the serious risk of brain trauma and infections (Lu et al., 2014). Non-invasive drug delivery strategies involve chemical or biological modification of target compounds which take advantage of various receptors present on the endothelial cells of the BBB. While promising, these methods are not scalable and face considerable regulatory hurdles (Pathan et al., 2009). Intranasal drug delivery is another noninvasive method of drug delivery that takes advantage of the direct olfactory nerve pathways to deliver drugs directly to the brain parenchyma (Crowe et al., 2018). However, it faces significant limitations including poor drug distribution, mucociliary clearance, and a paucity of olfactory neuroepithelial surface area. Additionally, the presence of various proteases and peptidases in the nasal mucosa can easily degrade the target molecules thereby affecting the amount of drug reaching the brain (Bleier et al., 2011; Touitou and Illum, 2013). Therefore, in spite of the promising nature of all these strategies, they are short lived and difficult to scale up to meet the needs of growing population affected by neurological disorders. Hence there remains a need for novel drug delivery techniques that can overcome these shortcomings and deliver drugs effectively to the brain.

Our group has previously reported on a novel method of permanently bypassing the BBB using an endoscopic heterotopic mucosal grafting technique within the nasal cavity (Pawar et al., 2018). This technique is based on established endoscopic skull base surgical techniques to repair the skull base following intradural approaches using mucosal grafts including the nasoseptal flap (Ishii et al., 2014; Miyake and Bleier, 2015a). These mucosal grafts have been shown to be 1000 times more permeable than BBB while remaining watertight and immunocompetent (Harvey et al., 2012; Miyake and Bleier, 2015a). Based on this significant permeability, our group has previously reported that they can be utilized to deliver high and low molecular weight drugs including recombinant neurotrophic factors directly to the brain (White et al., 2003; Hadad et al., 2006; Cassano and Felippu, 2009; Bleier et al., 2012, Bleier et al., 2013, Bleier et al., 2015; Harvey et al., 2012; Kimple et al., 2012; Chaaban and Woodworth, 2013).

In the current study we utilized an established rodent mucosal graft model previously developed by our group (Pawar et al., 2018) to determine the distribution and efficacy of transmucosal BDNF AT delivery in naïve rats and in a 6-OHDA model of PD. In order to further enhance the drug delivery and to protect the BDNF AT’s from nucleases, we also utilized a 200–250 nm cationic liposomal system.

In light of the potential benefits of BDNF AntagoNAT therapy in PD and the limitations for its delivery, we hypothesized that our heterotopic mucosal grafting approach could be utilized as a novel method for the direct CNS delivery of AntagoNAT’s.

## Materials and Methods

### Study Design

We used complementary *in vitro* (RT4-D6P2T rat schwannoma cells, *n* = 4–6 per condition) and *in vivo* (rat heterotopic mucosal grafting technique to bypass the BBB, *n* = 4–6 per treatment group) models to study the uptake, distribution, and efficacy of BDNF AntagoNATs (AT) in the derepression of BDNF transcription and protein expression. For *in vitro* studies, 3 treatment groups were used (negative control, AT in saline (AT-SALINE) and AT in liposomes (AT-LIPO)) with *n* = 4–6 per group. For studies in naïve rats, 4 treatment groups (negative control, positive control, AT in saline (AT-SALINE) and AT in liposomes (AT-LIPO) were used for qualitative studies and 3 treatment groups (negative control, AT in saline (AT-SALINE) and AT in liposomes (AT-LIPO) were used for quantitative studies with *n* = 4 per group. For studies in 6-OHDA rats, 3 treatment groups were used (vehicle + 6-OHDA, positive control and AT-LIPO + 6-OHDA) with *n* = 6 per group. For *in vivo* studies, two surgeries were performed on each rat (described in detail in the following section). First surgery involved implanting graft over the craniotomy and three days later a sterile propylene reservoir was placed over the graft to deliver required formulations. All rats were dosed with 0.15 mg/kg of AntagoNAT (cy5-BDNF-AT and BDNF-AT) in saline and liposomes three times every 72 h and were sacrificed 3 days after the last dose (for experimental timeline see Supplementary Figure S1). *In vitro* transfection efficiency and *in vivo* distribution was determined by histology using a Cy5 covalently labeled BDNF-AT (Cy5-BDNF-AT) and by hybridization assay using an unlabeled active BDNF-AT. In both models, a liposomal encapsulated formulation (AT-LIPO) was compared to a saline solution (AT-SALINE) and appropriate vehicle controls. *In vivo* therapeutic efficacy was tested in both healthy and in 6-OHDA PD rat models and compared to controls. The 6-OHDA treated rats were injected unilaterally in the right striatum and were dosed with 0.15 mg/kg of the required AntagoNAT formulation on the same day after installation of the propylene reservoir (three days after engrafting). Rats were dosed with 0.15 mg/kg of the formulation three times ever 72 h, and all rats were sacrificed sixteen days after 6-OHDA injection (for experimental timeline see Supplementary Figure S1).

### Statistical Analysis

All statistics were carried out using GraphPad Prism (version 7.00, La Jolla CA, United States, www.graphpad.com). Experimental data was analyzed by one-way and two-way ANOVA with post-hoc Tukey’s test for multiple comparisons (*p* value less than 0.05 was considered statistically significant).

### Animals

40 Male Sparague Dawley rats (250–300 g) were purchased from Charles River laboratories and used for the study. All animal procedures were approved by the Northeastern University Institutional Animal Care and Use Committee (Protocol number 180101-R). All surgeries were performed under isoflurane anesthesia and all efforts were made to minimize the suffering. All the rats were single housed in a climate-controlled room with 12/12-h light/dark cycle. All the rats were provided with food and water as required. A donor rat was used to harvest a free nasal septal mucosal graft according to the previously described methods (Bleier et al., 2013; Miyake and Bleier, 2015a). All rats were randomly assigned to control and treatment groups.

### Rat Heterotopic Mucosal Grafting Procedure

**FIGURE 1:**
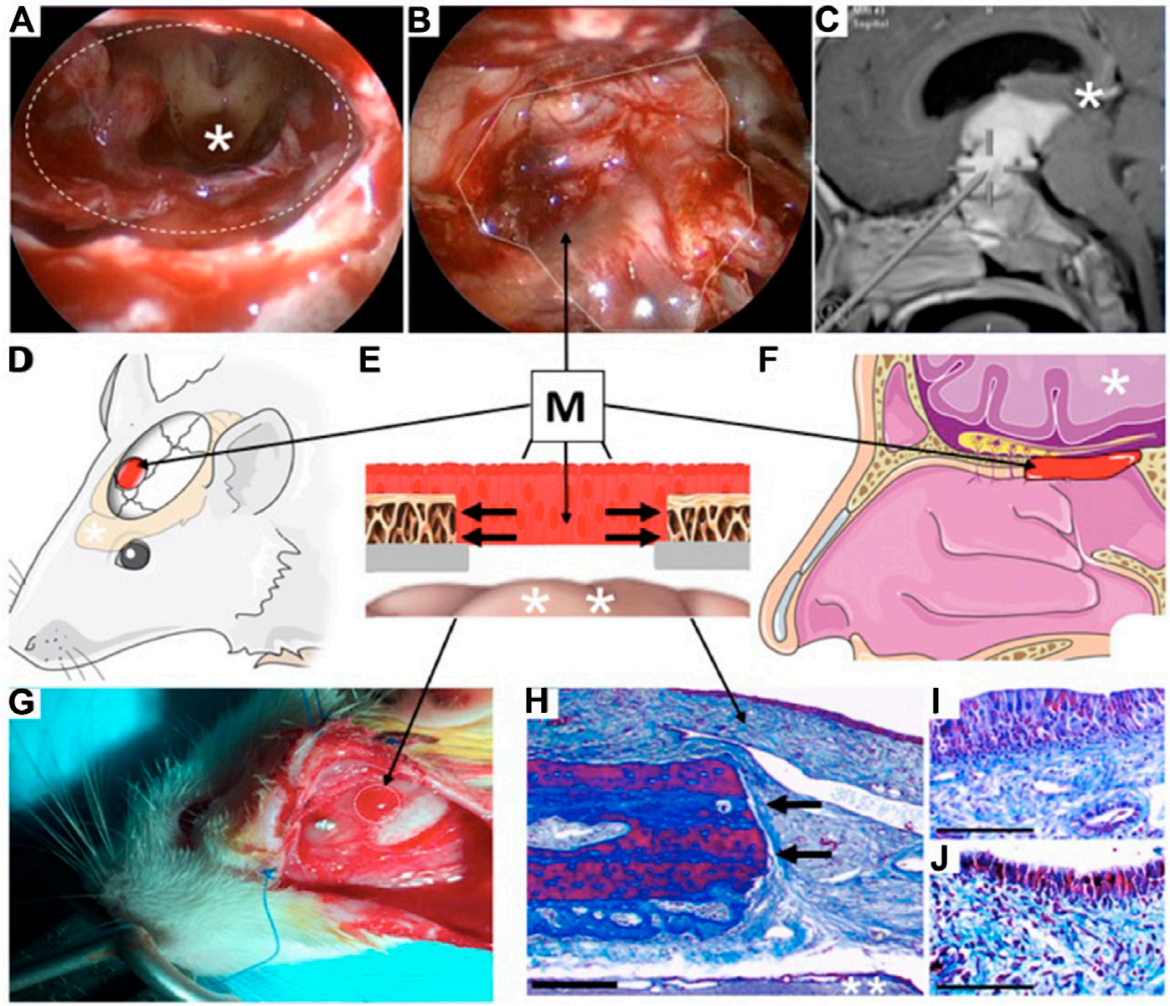
Clinical example and illustration of rodent heterotopic mucosal grafting technique. **(A)** Surgical endoscopic trans nasal view of human brain (*) through a skull base defect (dashed oval) demonstrating large communication between nasal cavity and central nervous system after tumor removal. **(B)** Reconstruction of surgical defect using a heterotopic mucosal graft (M, dotted line). **(C)** Trajectory of endoscopic approach through nostrils to reach brain (* represents same brain region seen in Figure 1A). **(D)** Illustration of rat model demonstrating location of mucosal graft (M) over the parietal lobe of the brain (*). **(E)** Cross sectional illustration of the human and rat skull after reconstruction demonstrating the mucosal graft (M) inset over the bony craniotomy (black arrows) as the only barrier between the external environment and the brain (*). **(F)** Illustration of position of mucosal graft (M) in a human nose demonstrating how the rodent model faithfully recapitulates the layers of human clinical skull base reconstruction. **(G)** Photograph of healthy engrafted mucosa in the rat model (M, dotted line). **(H)** Masson’s trichrome stain of the rat mucosal graft (M, bar = 20 μm) in same orientation as Figure 1E demonstrating healthy mucosa densely adherent to the cut edge of the bone (black arrows) with the intact underlying brain (**). **(I, J)**. High magnification views (Masson’s trichrome stain) of the graft epithelium on postoperative days 3 (Figure 1I) and 7 (Figure 1J) demonstrating intact and healthy mucosa at both time points (bar = 5 μm).

We used an innovative heterotopic mucosal engrafting technique to bypass the BBB and directly deliver BDNF-AT to the brain in male Sprague-Dawley rats. Briefly, a donor rat was euthanized by carbon dioxide and surgical scissors were used to remove the skin from the nasal dorsum. A surgical drill and scissors were used to remove and isolate a unilateral septal mucoperichondrial graft, which was stored in saline solution for a maximum of 2 h. The experimental rat (250–300 g) was anesthetized using isoflurane and placed in a stereotaxic frame under an operating microscope. The surgical site was sterilized using povidone iodine and alcohol swabs. A sagittal incision was made from the level of midorbit to the occiput using a scalpel. Bilateral skin flaps were then elevated exposing the pericranium. A scalpel was used to clear the pericranium from the intended craniotomy site (1.5 mm anterior-posterior and 2 mm medial-lateral to bregma). A surgical drill was used to create a 3 mm craniotomy leaving the underlying dura intact. The underlying dura and arachnoid were then removed leaving the underlying pia matter undisrupted. The harvested graft from the donor rat was then implanted over the craniotomy such that the basolateral membrane of the graft faced the exposed pia matter. Sterile nitrile was placed over the graft to prevent adhesion to surrounding tissue. The skin flaps were closed with suture and the rat was left to engraft for 3 days. After 3 days the skin flaps were again reflected carefully without disrupting the underlying implanted mucosal graft. The graft was then inspected using a dissection microscope to ensure viability and circumferential engraftment. A 250 µL polypropylene reservoir was then placed over the mucosal graft and attached to the skull using cyanoacrylate and tested for leaks using sterile saline. After implantation of a screw (Morris Precision screws and parts – 000 × 3/32 Flat self-tap screws), dental cement was applied to the skull to fix the reservoir. All rats were then dosed with 0.15 mg/kg of the formulations on the day the reservoir was instilled followed by two more doses every 72 h. All rats were then sacrificed three days after the last dose. Negative controls were considered rats who underwent a craniotomy without reflection of the dura and arachnoid which function as a blood-cerebrospinal fluid barrier. Positive controls were considered rats in which BDNF-AT was applied directly to the exposed pia after craniotomy and dura/arachnoid reflection without intervening graft placement (Figure 1).

### Masson’s Trichome Staining Protocol

Post-surgery, all rats were sacrificed using carbon-dioxide and whole rat heads were isolated. Each rat head was then placed in decalcifying solution for 24–48 h. Post decalcification, rat skulls were isolated and placed in 4% paraformaldehyde solution for 24–48 h. Samples were then refixed with Bouin’s solution for 1 h at 56°C to improve the staining quality. Each sample was then sectioned and stained using weigert’s iron hematoxylin working solution for 10 min. Sections were then rinsed in running warm tap water for 10 min and were then stained in Biebrich scarlet-acid fuchsin solution for 10–15 min. After washing in distilled water, sections were then differentiated in phosphomolybdic-phosphotungstic acid solution for 10–15 min. Sections were then transferred to aniline blue solution for 5–10 min. Sections were rinsed with distilled water and differentiated in 1% acetic acid solution for 2–5 min. After the last wash, sections were then dehydrated in alcohol and cleared in xylene. Sections were then mounted with resinous mounting medium and imaged on a microscope.

### 6-Hydroxydopamine Parkinson’s Disease Model in Rat

Following graft inspection 3 days after engrafting, rats were injected with 10 μL of 6-hydroxydopamine (6-OHDA) solution (10 μg/2 μL of 6-OHDA HBr salt (Sigma Aldrich 162957) in 0.1% ascorbic acid in saline) at the rate of 0.66 μL/min using a syringe pump [Harvard apparatus remote/infuse withdraw pump 11 elite nanomite programmable syringe pump (catalog no 70-4507)]. The solution was injected into the right striatum using a 5 μL neuros Hamilton syringe (catalog no 65460-03) at the following co-ordinates: anterior-posterior (AP), 0.5 mm; medial-lateral (ML), 2.5 mm; and dorsoventral (DV), 4.5 mm over 3 min. The syringe was kept in place for an additional 5 min after injection before withdrawal. After syringe withdrawal, the 250 μL propylene reservoir was implanted as described in the heterotopic grafting procedure section. Rats were then dosed with either saline (e.g., vehicle control) or 0.15 mg/kg of BDNF AT liposomes three times every 72 h (e.g., AT-LIPO). All rats were then sacrificed 16 days after 6-OHDA injection.

### Brain Derived Neurotrophic Factor AntagoNAT Design and Validation

Potential regulatory NATs in the BDNF locus on rat chromosome 3 were identified using the UCSC genome browser at https://genome.ucsc.edu/cgi-bin/hgGateway. The accession numbers of these transcripts were CN544668, AI030286, and BF391266. Single strand fully phosphorothiorated oligonucleotides (AntagoNATs) were designed to these three transcripts using the Oligo Design and Analysis Tools (IDT Inc., San Diego, CA, United States, at http://www.idtdna.com/calc/analyzer). The AntagoNAT sequences with no other significant homologies in the rat genome were selected. The AntagoNATs were then synthesized by IDT. Rattus norvegicus neuronal Schwann cell line RT4-D6P2T (ATCC# CRL-2768TM) was obtained from American Type Culture Collection (USA). RT4-D6P2T cells were cultured in EMEM with 10% of FBS and 1% of penicillin/streptomycin at 37°C and 5% CO_2_. AntagoNATs at 20 nM final concentration were transfected into RT4D6P2T cells using Lipofectamine™ 2000 (Life Technologies Corporation, Grand Island, NY, United States) as described in (Hsiao et al., 2016).

The total RNA was isolated using SV total RNA Isolation System (Promega, Madison, WI, United States) 48 h after transfection and the level of rat BDNF mRNA was quantified by real-time PCR using High Capacity cDNA Reverse Transcription Kit (Applied Biosystems, United States) and TaqMan gene expression mix with rat BDNF TaqMan Gene Expression Assay Rn02531967_s1 labeled with FAM. Results were normalized to 18S levels determined using Taqman 18S control assay labeled with VIC (Applied Biosystems catalog number 4319413E) and compared to BDNF levels in cells transfected with an inactive control oligonucleotide of similar chemistry and no homologies in the rat genome. Data analysis was performed using Excel software. The AntagoNAT C*A*T*A*G*G*A*G*A*C*C*C*T*C*C*G*C*A*A*C (termed BDNF-AT) against the CN544668 transcript showed the highest up-regulation of the BDNF mRNA levels and was selected for the remainder of the *in vitro* and *in vivo* experiments.

### Formulation and Characterization of Cy5-Brain Derived Neurotrophic Factor-AntagoNAT and Active Brain Derived Neurotrophic Factor-AntagoNAT Cationic Liposomes

The cationic lipid film was prepared using DOTAP [1,2 dioleoyl-3-trimethylammonium propane (chloride salt)] (a cationic lipid), cholesterol (stabilizer), and DPPC (1,2 Dipalmitoyl-sn-glycero-3-phosphocholine) (a neutral lipid) in a 5:3:5 Molar ratio. A stock concentration of each lipid was made in chloroform and 1 ml of each lipid was added to a 10 ml round-bottom flask attached to a Rotavap (IKA RV Control 10) and allowed to rotate at 100 rpm in a water bath at room temperature. When the chloroform had evaporated, the thin lipid film at the bottom of the flask was dried overnight in vacuum to remove solvent. The film was hydrated the next day, with 200 μg/ml of Cy5-BDNF-AT or active BDNF-AT in saline as a starting concentration followed by 1 min of vortexing. The liposomal preparation was placed on ice for 2 min, vortexed, placed in a water bath at 37°C for 2 min, and vortexed again. Five such freeze thaw cycles were performed. The preparation was then probe-sonicated for 5 min on ice. The mixture was ultracentrifuged in a Beckman-Coulter ultracentrifuge at 100,000 rpm for 1 h to separate the AT encapsulated liposomes from un-encapsulated AT. The pelleted liposomes were suspended in 400 µL of saline and extruded through 800 nm, 400 nm and 200 nm membranes, using an extruder (Avanti Lipids). The concentrated supernatant was used for further analysis. An indirect method was used to determine the encapsulation efficiency. The amount of AT in the supernatant was measured using nano drop and subtracted from the staring amount giving the total AT encapsulated in liposomes. Extruded liposomes were characterized for size, PDI (Polydispersity index) and charge, using a Zetasizer (Nano-ZS90, Malvern Instruments, Inc. Westborough, MA, United States). Liposomal morphology was determined using transmission electronic microscopy (TEM). The liposomal particles were negatively stained using 1.5% uranyl acetate. A 300-mesh carbon coated copper grid was applied to 10 μL of liposomal sample (10x diluted). Excess sample was wicked off by briefly touching the grid to a piece of filter paper followed by 3 brief DI water rinses followed by touching the grid to 1.5% uranyl acetate stain 3–5 times and wicking off the excess fluid after each step. Sample grids were viewed using a JEOL, JEM 1010 TEM operated at 80 kV.

### Qualitative Evaluation of Cy5-Brain Derived Neurotrophic Factor-AntagoNAT Uptake in RT4-D6P2T Rat Schwannoma Cell Line

The RT4-D6P2T rat schwannoma cells (American type culture collection) were incubated at 37°C with 5% CO_2_ in DMEM with 10% FBS and 2% antibiotics. All exposures occurred at 80% confluency. Cells were seeded into 6 well plates at a density of 2 × 10^5^ cells per well and were cultured in 2 ml of FBS free DMEM for 24 h at 37°C with 5% CO_2_. Cells were then incubated with 300 nM of Cy5-BDNF-AT in saline and cationic liposomes for 30 min, 4 h, 8 h and 12 h. After incubating the cells with Cy5-BDNF-AT for specified time points the cells were washed twice with 1X PBS and were fixed with 4% formalin for 10 min. A drop of DAPI stain was then added to each slide before placing the coverslips for nuclei staining. After DAPI staining, the coverslip was glued to the slides and the slides were further observed under confocal microscope for determining the uptake in cells (Zeiss LSM 700 laser scanning confocal microscope, Thornwood, NY, United States, Magnification used—10x, all images were taken using auto exposure settings from the ZEN 2009 software).

### Quantitative Evaluation of Active Brain Derived Neurotrophic Factor-AntagoNAT Uptake in RT4-D6P2T Rat Schwannoma Cell Line

Quantitative transfection studies were done in RT4-D6P2T rat schwannoma cells. Cells were seeded as described above and treated with 50, 100, and 300 nM of active BDNF-AT in saline and cationic liposomes for 12, 24, and 48 h. A hybridization assay was developed to detect the concentration of BDNF-AT in samples. Cells were washed with 1X PBS, pelleted, and lyzed using the RIPA lysis buffer (Thermo Fisher Scientific) containing the EDTA-free protease inhibitor cocktail (Roche). The cell lysate was then centrifuged at 20,000 ×*g* for 20 min to remove cell debris. The supernatant was then used in the hybridization assay as follows. The capture and detection probes were designed to specifically detect BDNF-AT. The capture probe was complementary to the 3′ end and the detection probe was complementary to the 5′ end of the BDNF-AT as follows:

BDNF-AT sequence - 5′-C*A*T*A*G*G*A*G*A*C*C*C*T*C*C*G*C*A*A*C-3′
Capture probe - (5AmMC12//iSp18/iSp18//G*+T*+T*+G*+C*+G*+G*+A*+G).
Detection probe - (+G*+G*+T*+C*+T*+C*+C*+T*+A*+T*+G/iSp18//iSp18//iBiodT//3BioTEG).

where * designates phosphorothioate bond, + designates LNA modifications, 5AMmc12 is a 5′-amino modifier C12m, iSp18 is an internal 18-mer spacer, iBiodT is an internal biotin-dT and 3BioTEG is a 3′ biotin-TEG). The probes were synthesized by Qiagen Inc., Germantown, MD, United States, 20,874–1415 (capture probe catalog no: 339412 YCO0070251 and detection probe catalog no: 339412 YCO0070253). The capture and detection probes were reconstituted with nuclease-free water to 5000 pmol/ml. 40 μl of the 5000 pmol/ml capture probe was then added to 19.96 ml of 500 mM Na_2_HPO_4_ and 1 mM Na_2_EDTA (pH 8.5). 150 μL of the capture probe mix was added to each well of a 96-well white Nunc™ plate (Thermo scientific, catalog no 436007) and incubated overnight at 4°C. The next day the coated plate was washed five times with 300 μL/well of wash buffer (1X TBST). After the last wash the plate was incubated with blocking buffer (3% BSA in 1X DPBS) for 2 h at room temperature on a plate shaker and washed. Then 150 μL of the detection probe/sample mix was added to each well. The detection probe/sample mix was prepared as follows. First, 200 μL of 5000 pmol/ml of detection probe was added to 19.8 ml of 4X SSC/0.5% sarkosyl buffer. Then the diluted detection probe (225 μL) and the cell extract sample (25 μL) were annealed in a thin walled V bottom plate using a thermocycler (RT 100 Bio-Rad) at 90°C for 12.5 min followed by incubation at 40°C. After addition of annealed product, the coated plates were incubated at 45°C for 2 h on a plate shaker. Then the plates were washed again and incubated with diluted streptavidin-HRP conjugate (Jackson Immunoresearch) (1:50,000 times diluted with poly HRP buffer (Thermo Fisher Scientific) for 30 min at 37°C on a plate shaker. The plate was washed again, and the luminescence was detected immediately by adding 150 μL of the Elisa Femto Solution Mix (Thermo Scientific).

### Quantification of *in vitro* Brain Derived Neurotrophic Factor-AntagoNAT Mediated Brain Derived Neurotrophic Factor mRNA Derepression

After harvesting the BDNF-AT exposed RT4-D6P2T cells as previously described, cells were lyzed and processed for RNA extraction using Quick-RNA™ MiniPrep kit (Zymo Research). The extracted RNA was used for cDNA synthesis using the SuperScript™ IV VILO™ Master Mix (Thermo Fisher Scientific). qPCR was then performed using the TaqMan™ Fast Advanced Master Mix (Thermo Fisher Scientific) according to manufacturer’s instructions using the LightCycler 480 Instrument II as a PCR platform. The 18S RNA was used as an internal control.

### Quantification of *in vitro* Brain Derived Neurotrophic Factor-AntagoNAT Mediated Brain Derived Neurotrophic Factor Protein Derepression

After harvesting the BDNF-AT exposed RT4-D6P2T cells as previously described, cells were washed with 1X PBS, pelleted, and lyzed using the RIPA lysis buffer (Thermo Fisher Scientific) containing the EDTA-free protease inhibitor cocktail. The cells were incubated with the lysis buffer for 15 min and the cell lysate was then centrifuged at 20,000 g for 20 min to extract the protein. The supernatant (protein) was then collected, and samples were diluted 6x to detect the BDNF levels using a commercially available BDNF ELISA kit (EMD Millipore).

### Quantification of *in vitro* Brain Derived Neurotrophic Factor-AntagoNAT Mediated Cytotoxicity in RT4-D6P2T Rat Schwannoma Cells

RT4 Schwannoma cells were seeded at a density of 8000 cells per well in a 96 well flat bottom cell culture plate (Denville Scientific Inc.) and cultured in 200 µL of growth medium for 24 h at 37°C with 5% carbon dioxide. After RT4-D6P2T cell exposures to BDNF-AT as described above, the old media was replaced by fresh growth media containing the blue stain (Hoechst 33,342 for live cells) and green stain (propidium iodide for dead cells) from the ReadyProbes™ Cell Viability Imaging kit (Molecular probes). The 96-well plate was then incubated for 15 min and each well was imaged using Keyence BZ-X710 All-in-One Fluorescence microscope. The % of viable cells in each well were quantified using ImageJ software.

### Qualitative Evaluation of Transmucosal Cy5-Brain Derived Neurotrophic Factor-AntagoNAT Distribution in the Rat Brain

Cy5-BDNF-AT in saline and cationic liposomes were delivered to the rat brain through the mucosal graft following verification of successful engraftment as described above. The implanted reservoir was filled with 100 µL of Cy5-BDNF-AT in saline and cationic liposomes for a total dose of 0.15 mg/kg. Rats were then re-dosed every 72 h for a total of 3 times. Positive (direct parenchymal dosing) and negative (craniotomy without dura/arachnoid reflection) controls were additionally dosed with Cy5-BDNF-AT in saline at the same concentration and dose regimen. All rats were sacrificed 3 days after the last dose (e.g., 12 days after the initial dose). Rats were sacrificed by administering carbon-dioxide and brains were immediately dissected from the skull of each rat. All extracted brains were immediately flash frozen in acetone and dry ice solution, and placed in OCT solution −80°C. Brains were sliced into 50 micron sections using a cryotome and the sections comprising the striatum, hippocampus and substantia nigra were imaged under an epifluorescence microscope (Leica DM IL LED fluorescent microscope (Leica, Buffalo Grove, IL, United States) (Magnification ×20, exposure 229.4 ms, Cy5 filter gain settings for striatum, hippocampus and substantia nigra were 2, 5.5, and 8 respectively). The total cy5-flourescence intensity for each image was quantified using ImageJ software by employing the equation Integrated density = [area of selected region (fluorescent region) × mean fluorescence of the selected area] − background mean fluorescence.

### Quantitative Evaluation of Transmucosal Active Brain Derived Neurotrophic Factor-AntagoNAT Distribution in the Rat Brain

Active BDNF-AT in saline and cationic liposomes were delivered to rat brain through the mucosal graft as described above. The implanted reservoir was filled with 100 µL of BDNF-AT in saline and cationic liposomes for a total dose of 0.15 mg/kg. Rats were then re-dosed every 72 h for a total of 3 times. Negative controls as described above were additionally dosed with BDNF-AT in saline at the same concentration and dose regimen. Rats were sacrificed by administering carbon-dioxide. Following sacrifice, rat brains and olfactory bulbs were immediately isolated, and 3 mm tissue biopsy punches (Integra miltex) were used to isolate the striatum, hippocampus, substantia nigra and cerebellum both ipsilateral and contralateral to the side of the mucosal graft dosing site. The tissue punches were homogenized in 300 μL ice cold tissue lysis buffer and homogenates were centrifuged at 20,000 g for 20 min to extract the total protein. The extracted protein was then used for the BDNF-AT hybridization assay as described above. The detected AT levels were then normalized to the total protein content in the samples measured using a Pierce BCA assay kit (Thermo Fisher scientific).

### Quantification of *in vivo* Brain Derived Neurotrophic Factor-AntagoNAT Mediated Brain Derived Neurotrophic Factor Protein Derepression in the Rat Brain

A portion of the extracted protein samples isolated during the BDNF-AT hybridization procedure mentioned above were used to quantify BDNF protein levels in each of the isolated tissue punches. BDNF protein concentrations were determined by commercially available ELISA (EMD Millipore CYT306) and normalized to the total protein content in the sample measured using the Pierce BCA assay kit. The data was reported as % of control normalized to protein content.

### Quantification of Tyrosine Hydroxylase and Brain Derived Neurotrophic Factor in 6-OHDA Parkinson’s Disease Rat brains using Western Blot

After sacrificing PD rats sixteen days after 6-OHDA injection, rat brains were isolated, and 3 mm biopsy punches were used to isolate striatum and substantia nigra. The tissues were then homogenized with 220 μL of ice-cold pierce RIPA lysis buffer and the tissue homogenate was centrifuged at 12,000 g for 20 min at 4°C to extract total protein. Total protein was quantified using pierce BCA assay kit (catalog no 23225) and 50 μg protein of each sample was loaded into each well of 4–12% Bis tris gels and proteins were separated using XCell SureLock mini cell electrophoresis system. The separated proteins were then transferred into PVDF membrane using iBlot from Invitrogen. The PVDF membrane with the transferred protein was blocked in 5% milk in 1X TBST for 1 h and then the blots were incubated overnight with Tyrosine hydroxylase (TH antibody (F-11): catalog no sc-25269, 1:1000), BDNF (anti-BDNF antibody (3C11) catalog no ab203573, 5 μg/ml) and β-actin (β-actin (C4) antibody: catalog no sc-47778, 1:200) primary antibodies at 4°C on a shaker. The next day the blots were washed with 1X TBST for 45 min and were incubated in *m*-IgGκ BP-HRP (catalog no 516102, 1:1000 for santa cruz primary antibodies) and goat anti-mouse IgG (H + L) HRP secondary antibody (catalog no ab 97,023 for abcam primary antibody) secondary antibodies for 2 h at room temperature on a shaker. The membranes were then washed using 1X TBST for 45 min and the blots were then developed using enhanced chemiluminescent detection reagent (thermo fisher scientific catalog no 34580 and Santa Cruz biotechnology catalog no sc-2048). The blots were then imaged using BioRad molecular imager ChemiDOX™ XRS + imaging system and a semi-quantitative analysis was performed on the blots using ImageJ. Results are expressed as TH and BDNF protein band intensity normalized to β-actin band intensity.

### Immunohistochemical Analysis of Tyrosine Hydroxylase Expression in the Rat Brain

Rats were sacrificed sixteen days after 6-OHDA injection and were transcardially perfused with 200 ml of 1X PBS and then with 200 ml of 4% paraformaldehyde (Sigma catalog no 1004965000). The brains were then post-fixed with 4% paraformaldehyde for 4 h and then were transferred into 30% sucrose solution in 1X PBS for 24 h at 4°C. The brains were then embedded in reservoirs using optimal cutting solution (OCT). The next day rat brains were sectioned into 50 μm thick coronal slices and the brain slices were stored in 0.1% sodium azide in 1X PBS until staining. The sections were washed for 30 min in 0.5% triton X-100 and 100 nmol/L glycine in PBS and then in PBS containing Triton X-100 for 1 h (20 min 3 times). The sections were then blocked in UltraCruz blocking reagent (catalog no sc-516214) for 2 h at room temperature. Sections were incubated overnight in TH primary antibody diluted in blocking reagent (TH antibody (F-11): catalog no sc-25269, 1:50) at 4°C on a shaker. The next day the sections were washed in PBS containing Triton X-100 (20 min 3 times) and then were incubated in *m*-IgGκ BP-CFL 488 secondary antibody (catalog no sc-516175, 1:50) at room temperature. Sections were then washed in PBS containing Triton X-100 (20 min 3 times) and in 1X PBS (20 min 3 times). Sections were then mounted on glass slides using vectashield mounting medium (Vector Laboratories H-1000). The coverslips were secured in places on the slides using a transparent nail polish. The images were captured using Keyence B2-X710 all-in-one fluorescence microscope.

## Results

### Brain Derived Neurotrophic Factor AntagoNAT and Surgical Validation

Three transcripts that represented potential regulatory NATs were identified in the BDNF locus on rat chromosome 3 using the UCSC genome browser at https://genome.ucsc.edu/cgi-bin/hgGateway. One of the AT’s designed against the CN544668 transcript that consistently showed significant upregulation of the BDNF mRNA levels (See Supplementary Figure S2) was named BDNF-AT and selected for the *in vivo* and *in vitro* experiments. All rats tolerated the surgical procedure with no attrition. There was complete mucosal engraftment in all rats with no evidence of graft malposition or necrosis (Figure 1).

### *In vitro* Uptake and Efficacy in RT4-D6P2T Rat Schwannoma Cells

**FIGURE 2:**
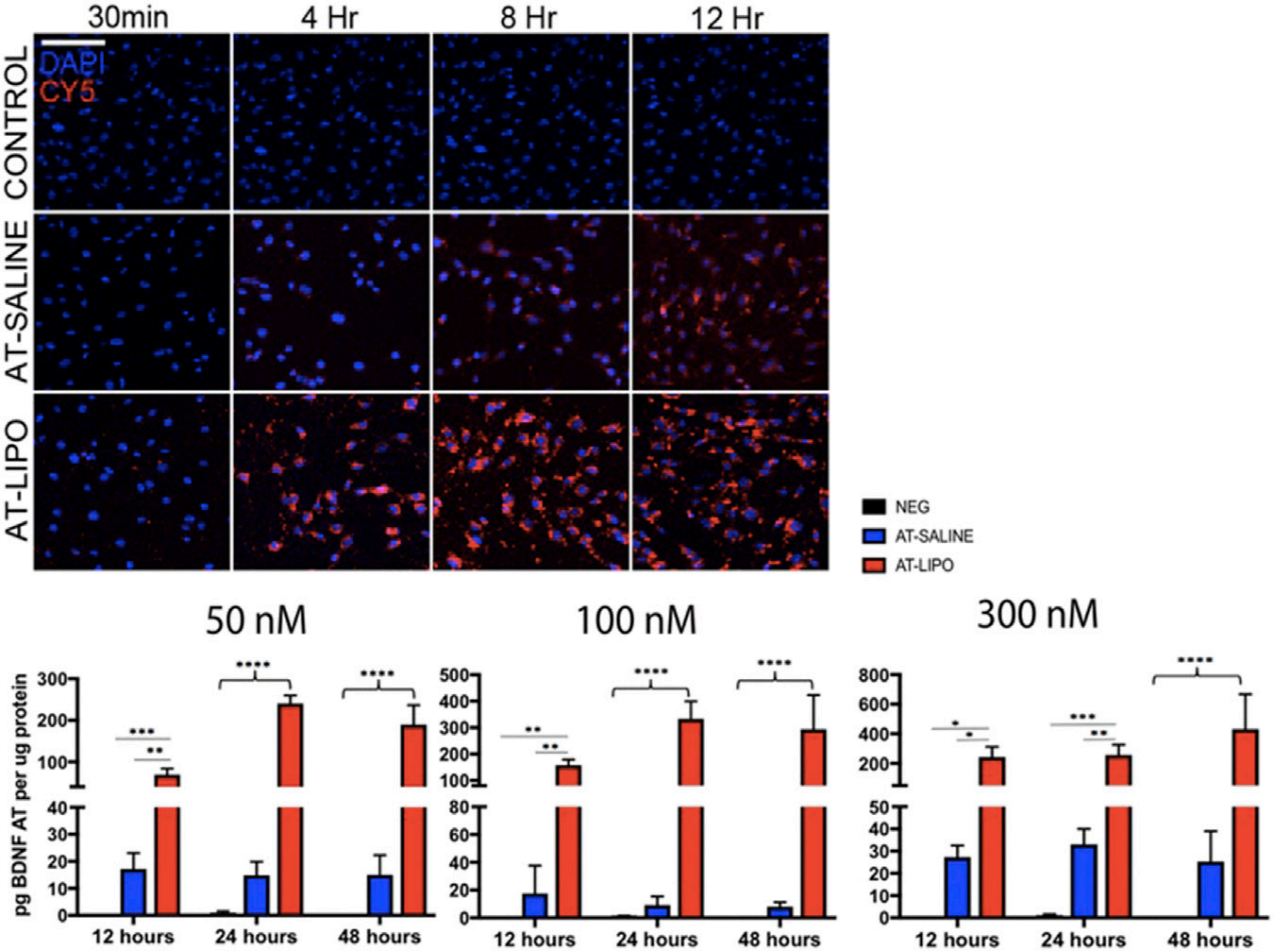
Evaluation of in vitro uptake of Cy5-BDNF-AT and BDNF AT cationic liposomes and saline formulation in RT4-D6P2T rat schwannoma cells using confocal microscopy and AT hybridization assay. Confocal microscopic images of time dependent uptake of vehicle control and Cy5-BDNF-AT at 300 nM in RT4-D6P2T rat schwannoma cells demonstrating more rapid and robust transfection in the liposomal (AT-LIPO) vs. saline (AT-SALINE) group (bar = 500 μm). Histograms represent quantification of active BDNF-AT uptake by dose, time, and formulation in the RT4-D6P2T cell line relative to protein normalized control (NEG represents vehicle control). Note the significant dose dependent increase in uptake efficiency in the liposome relative to the saline and negative control groups (*p < 0.05, **p < 0.01, ***p < 0.001, ****p < 0.0001; two-way ANOVA). Data are presented as mean ± SD (*n* = 4–6).

**FIGURE 3:**
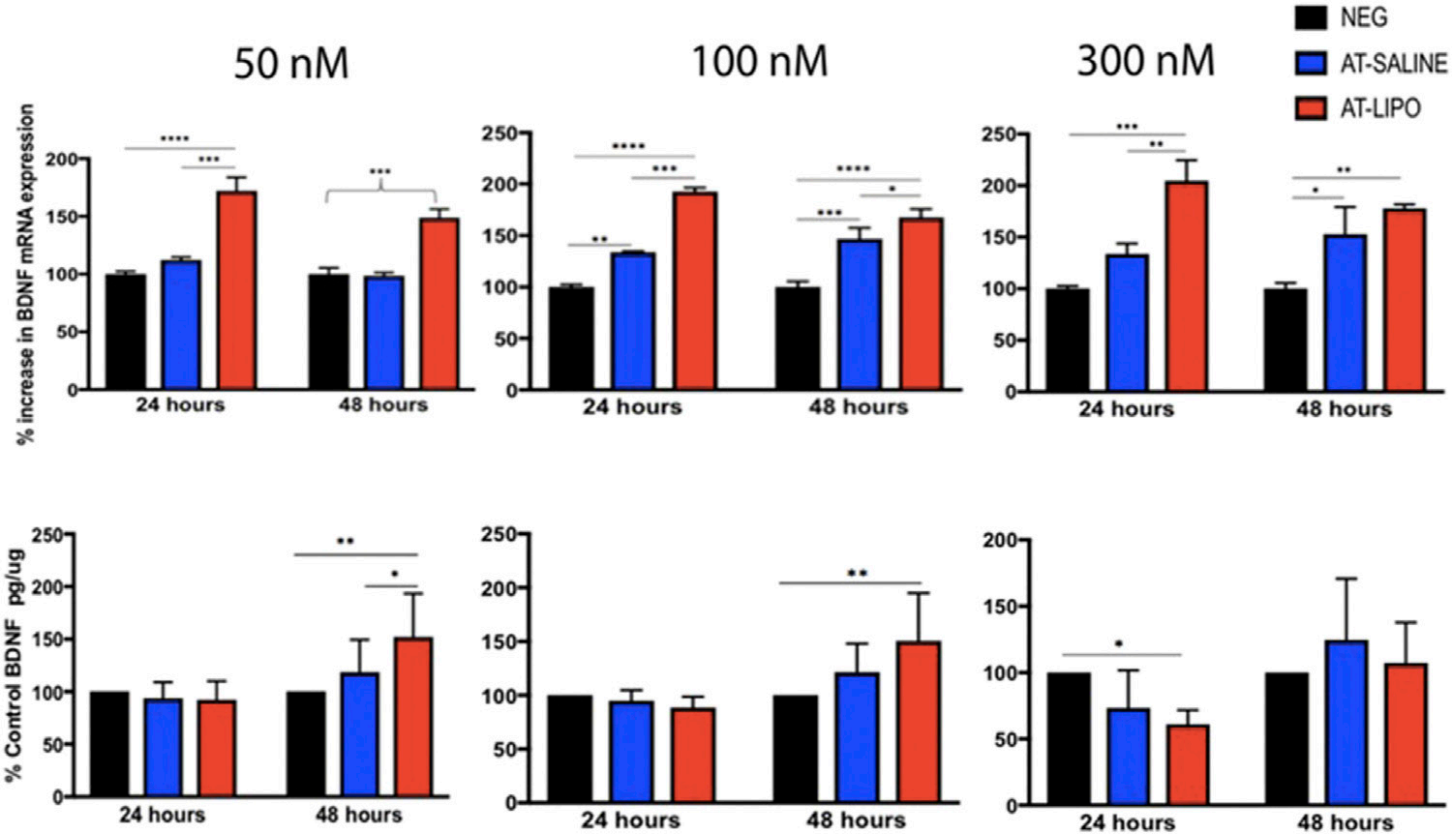
Evaluation of in vitro transfection efficiency of BDNF AT cationic liposomes and saline formulation in RT4-D6P2T rat schwannoma cells using qPCR and BDNF specific ELISA. Bar graphs of qPCR (top row) and ELISA (bottom row) demonstrating BDNF transcriptional and protein expression in RT4-D6P2T rat schwannoma cells following exposure to vehicle control (NEG), liposomal (AT-LIPO), and saline (AT-SALINE) BDNF-AT demonstrating both a dose and time dependent upregulation among the 50 and 100 nM concentrations (*p < 0.05, **p < 0.01, ***p < 0.001, ****p < 0.0001; two-way ANOVA). Data are presented as mean ± SD (*n* = 4 for qPCR analysis and *n* = 6 for ELISA).

The blank and BDNF-AT cationic liposomes had an average size of 206.5 ± 0.0 and 229.4 ± 17.6 nm, respectively (size obtained from zetasizer). A 100% encapsulation efficiency was achieved (See Supplementary Figure S3). Using confocal microscopy, the liposome-encapsulated 300 nM Cy5-BDNF-ATs demonstrated qualitative uptake as early as 30 min after exposure while the 300 nM Cy5-BDNF-AT uptake was delayed until 4–8 h (Figure 2). Quantification of transfection efficiency by AT hybridization assay between 12 and 48 h exhibited a dose response with less than 20% cytotoxicity from 50 to 300 nM (See Supplementary Figure S4). The liposome-encapsulated AT demonstrated significantly greater uptake at all doses and time points relative to the saline AT (Figure 2). Similarly, qPCR revealed that the liposomal group demonstrated significantly greater dose and time dependent BDNF transcription than the saline group. BDNF protein levels followed a similar pattern for the 50 and 100 nM dosing conditions while the increased expression among the 300 nM conditions was not statistically significant (Figure 3).

### *In vivo* Transmucosal Distribution in the Rat Heterotopic Mucosal Graft Model

**FIGURE 4:**
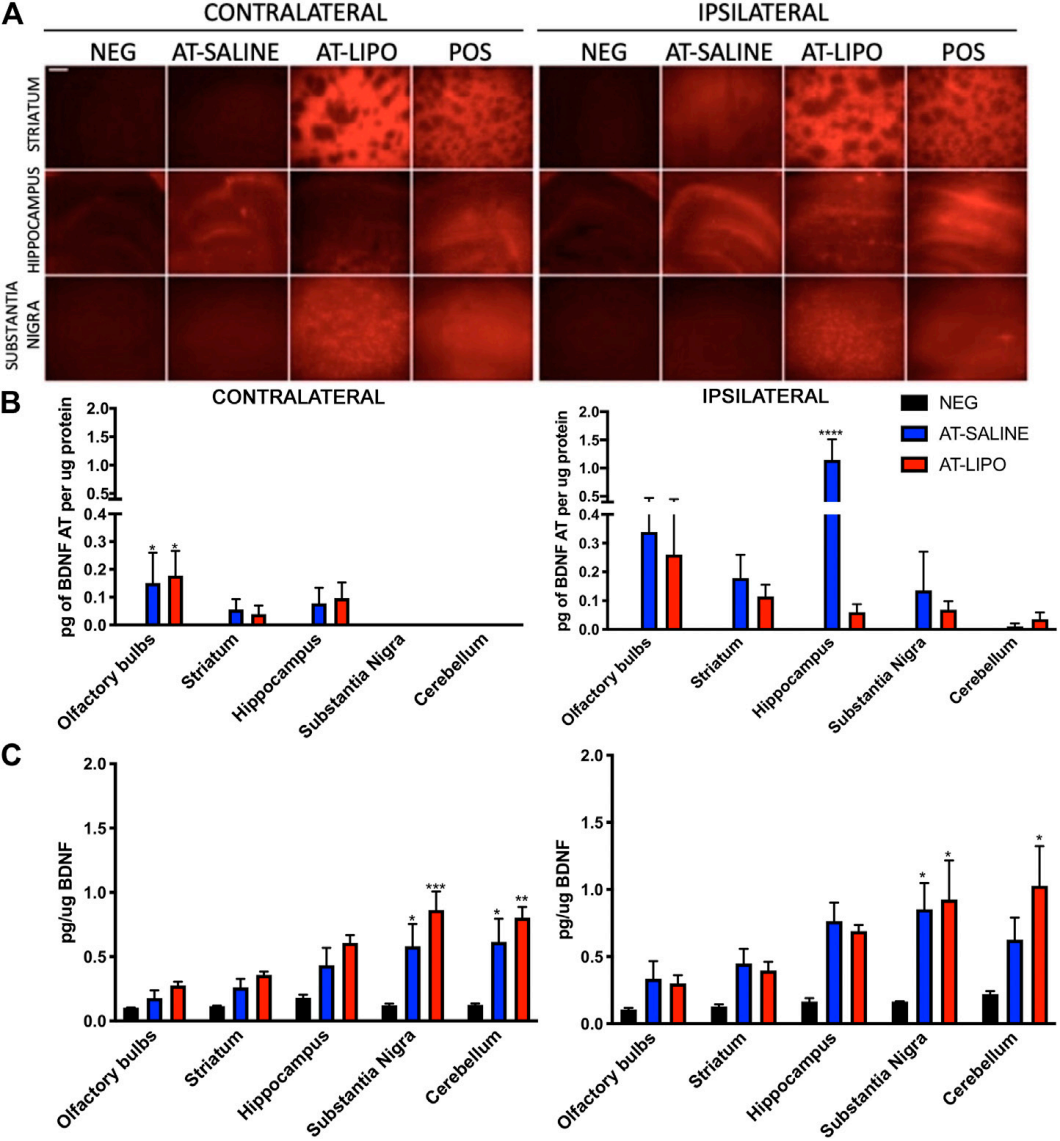
*In vivo* qualitative and quantitative transmucosal uptake and efficacy of BDNF AT (cy5-labeled and active) in saline and cationic liposomal formulation. **(A)** Fluorescent microscopic images of liposomal (AT-LIPO) and saline (AT-SALINE) formulation of Cy5-BDNF-AT distribution on post-dosing day 12 (scale bar = 500 μm demonstrating Cy5 distribution by side and brain end-target sub-region. **(B)** Bar graphs quantifying BDNF-AT by hybridization assay in the ipsilateral and contralateral end-target regions in among the liposomal (AT-LIPO) and saline (AT-SALINE) groups (****, *p* < 0.0001). **(C)** BDNF-AT derepressed protein expression by side and delivery vehicle group in the same end target sub-regions of the rat brain (*p < 0.05, **p < 0.01, ***p < 0.001; two-way ANOVA). Data are presented as mean ± SD (*n* = 4).

Following sacrifice 12 days after the initial transmucosal dose, the Cy5-BDNF-AT in saline group demonstrated a general trend toward increased distribution in the hemisphere ipsilateral to the mucosal graft relative to the contralateral side. Conversely, the liposomal Cy5-BDNF-AT group trended toward improved distribution on the contralateral side. Among the striatum and substantia nigra region, the liposomal group demonstrated significantly greater distribution than both the negative and positive control on both sides. Within the hippocampus, the saline group had significantly greater distribution than both the negative control and liposomal group (Figure 4A).

We next utilized a hybridization assay to quantify the distribution of both the liposomal and saline BDNF-AT formulations in the relevant brain end-target regions. No BDNF-AT was detectable within the negative controls. In general, there was greater BDNF-AT in the hemisphere ipsilateral to the mucosal graft regardless of group. There were no significant differences in distribution between liposomal and saline BDNF-AT formulations with the exception of the ipsilateral hippocampus where the saline group outperformed the liposomal group (Figure 4B).

### *In vivo* Transmucosal Efficacy in the Rat Heterotopic Mucosal Graft Model

In order to test the functional efficacy of the BDNF-AT, we then quantified BDNF protein expression in both groups relative to basal expression in control brains. These results demonstrated a trend toward increased expression in all brain sub-regions regardless of side or delivery vehicle although there were no strong correlations between BDNF AT levels and expression. The substantia nigra demonstrated significant BDNF upregulation relative to control bilaterally among both the liposomal and saline formulations while cortical regions demonstrated upregulation ipsilaterally only. Though not statistically significant, PD relevant region striatum also demonstrated BDNF protein up regulation bilaterally relative to the control group (Figure 4C, See Supplementary Figure S5).

### *In vivo* Transmucosal Efficacy in the Rat 6-hydroxydopamine Heterotopic Mucosal Graft Model of Parkinson’s Disease

**FIGURE 5:**
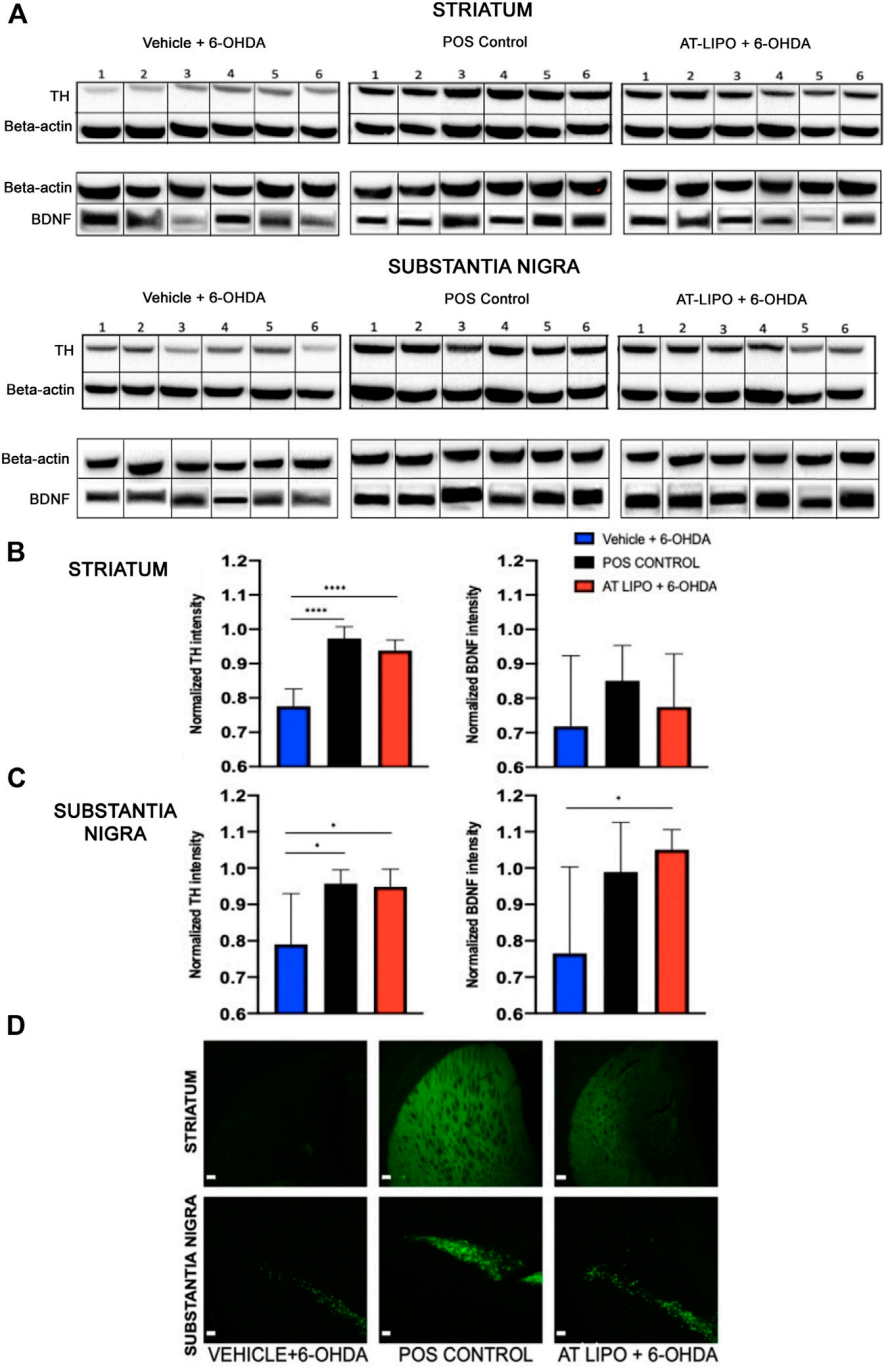
Therapeutic efficacy of AT-LIPO in rat 6-OHDA injury model. **(A)** Representative western blot images for TH and BDNF proteins in striatum and substantia nigra in 6-OHDA PD model rats by treatment group. **(B)** TH and BDNF expression by western blot within rat striatum demonstrating significant preservation of TH expression following AT-LIPO treatment (****, *p* < 0.0001, one-way anova, values are normalized to β-actin) and a non-significant trend toward BDNF preservation. **(C)** TH and BDNF expression by western blot within rat substantia nigra demonstrating significant preservation of both TH and BDNF expression following AT-LIPO treatment (*, *p* < 0.05, one-way anova, values are normalized to β-actin) relative to vehicle control. Data represented as mean ± SD (*n* = 6). D) Coronal fluorescent IHC images of TH staining in the rat 6-OHDA injury model (ipsilateral to injection, bar = 200 μM) demonstrating qualitative preservation of TH immunopositivity in both the striatum and substantia nigra following AT-LIPO treatment relative to vehicle control and positive control (POS control represents baseline expression in uninjured rat).

In order to determine the therapeutic efficacy of the liposomal BDNF -AT (AT-LIPO), we next examined both tyrosine hydroxylase (TH) and BDNF protein expression within the striatum and substantia nigra in an established 6-OHDA rat model of PD (Rabie et al., 2018). We demonstrated that BDNF AT’s encapsulated in liposomes (AT-LIPO) were associated with preservation of both TH and BDNF protein expression measured quantitatively using western blot and qualitatively using immunohistochemical (IHC) techniques relative to vehicle control. These results suggested that BDNF AT’s delivered using liposomes conferred a neuroprotective effect in the 6-OHDA induced disease state (Figure 5).

## Discussion

BDNF has been identified as a key target in PD as both BDNF protein and mRNA expression are reduced in patients with PD (Mogi et al., 1999; Parain et al., 1999). Treatment with BDNF has been shown to prevent the loss of dopaminergic neurons in the substantia nigra after a 6-hydroxydopamine or 1-methyl-4-phenylpyridinium (MPP)-induced lesion in rodents (Frim et al., 1994; Levivier et al., 1995). BDNF protein infusion has also exhibited therapeutic anatomical and behavioral effects in an MPP primate model of PD by reducing dopaminergic cell loss and enhancing striatal reinnervation (Tsukahara et al., 1995). However off-target toxicities associated with recombinant neurotrophic factor delivery (Nutt et al., 2003) have limited its clinical adoption.

In order to enhance BDNF expression while overcoming the limitations of recombinant BDNF protein delivery, we chose to target the endogenous regulatory mechanism mediated by a BDNF locus-specific non-coding RNA from the natural antisense transcript (NAT) class. We showed that inhibiting the activity of the rat BDNF NAT using oligonucleotide-based compounds (AntagoNATs) resulted in upregulation of BDNF mRNA *in vitro*. As AntagoNATs upregulate the endogenous protein, the problems of host protein contamination, inappropriate post-transcriptional modifications and subcellular localization that lead to recombinant protein toxicity are avoided. Chemically AntagoNATs are designed to rectify poor stability in biological matrices and interactions with the immune system which plagued the earlier versions of oligonucleotide drugs. Furthermore, AntagoNATs are designed to be taken up into cells directly thereby negating the need for viral or other carriers which are frequently toxic and can be immunogenic (Geary et al., 2015). Consequently, the use of BDNF-derepressing AntagoNATs has significant therapeutic potential in PD (Mogi et al., 1999; Parain et al., 1999; Magistri et al., 2012; Modarresi et al., 2012; Wahlestedt, 2013; Halley et al., 2014).

We utilized a cationic liposomal system to enhance drug delivery to the rat brain and to protect BDNF AT’s from nucleases. Liposomes are small artificial spheres made of cholesterol and phospholipids that can encapsulate both hydrophilic and hydrophobic drug molecules (Akbarzadeh et al., 2013). Several other drug delivery systems including aptamers-mediated delivery, exosomes, cell-penetrating peptides are currently being studied as potential CNS drug delivery systems (Bukari et al., 2020). They have the advantage of exploiting endogenous transport mechanisms on the blood-brain barrier for effective drug delivery into the brain (Bukari et al., 2020). For example, aptamers are RNA or DNA sequences that are highly target specific and have improved properties when compared to antibodies. Due to their small size and high binding affinities, they can penetrate cells, tissues and barriers (e.g., BBB) efficiently (Ashrafuzzaman, 2014; Bukari et al., 2020). Currently exosomes are also being explored as potential drug delivery systems owing to their small size (30–150 nm), low immunogenicity, stability, biocompatibility and efficient uptake into cells (Zheng et al., 2019). In comparison to these recently explored delivery systems, liposomes also offer several advantages like stability, increased efficacy, biodegradability, biocompatibility, reduced toxicity and sustained drug release (Akbarzadeh et al., 2013). We utilized cationic liposomes for this study as they can encapsulate AT’s efficiently owing to the negative charge.

In 2014 Claes Wahlestedt et al. showed that BDNF AntagoNAT’s were capable of significant upregulation of BDNF mRNA in a concentration-dependent manner in mouse N2a cell line (Modarresi et al., 2012). Our study validated this work by determining the transfection efficiency of cationic liposomes encapsulating BDNF AT in RT4-D6P2T rat schwannoma cells as compared to only BDNF AT in saline. The *in vitro* data confirmed that our BDNF AntagoNAT constructs encapsulated in liposomes are capable of inducing both BDNF transcription and translation in BDNF NAT-expressing rat schwannoma cells at 48 h. Though BDNF AT in saline did show some BDNF mRNA and protein upregulation, our study confirmed that the liposomal formulation was capable of enhancing both transfection and efficacy significantly over the saline vehicle alone.

Having demonstrated that BDNF-ATs could successfully upregulate BDNF *in vitro*, we next evaluated their *in vivo* distribution and efficacy in naïve rats. Prior to this, Wahlestedt et al. (Modarresi et al., 2012) utilized intracerebroventricular (ICV) delivery of BDNF AT’s to healthy C57BL6 mice and showed that continuous administration of BDNF AT for 28 days resulted in significant increase in BDNF mRNA levels and protein levels in mice. In our study we adopted a novel transmucosal delivery strategy previously described by our group (Miyake and Bleier, 2015b) which is capable of delivering molecules up to 500 kDa directly to the brain. This method is currently used by surgeons to reconstruct skull base defects after endoscopic endonasal removal of brain tumors. The technique involves the intranasal replacement of a segment of arachnoid and dura with highly permeable vascularized nasal mucosa which can then be used to deliver therapeutic agents directly to the CNS (Bleier, 2012). Heterotopic mucosal grafting offers several advantages over invasive BBB penetrating methods such as intrathecal delivery. First, this technique provides a permanent method of bypassing the BBB using only autologous tissue. Second, this approach has a long track record of clinical safety ensuring a permanent conduit for drug delivery without incurring a significant risk of infection, CSF leak, or meningitis (Bleier et al., 2012; Chaaban and Woodworth, 2013). Third, endoscopic endonasal mucosal grafting is widely performed using existing surgical instrumentation which is nearly universally present in modern operating rooms. Finally, the most conservative estimates suggest that the grafting method is less than half the cost of invasive catheter based CNS delivery techniques (Centers for Medicare and Medicaid Services Physician Fee Schedule; Gill et al., 2003; Al-Tamimi et al., 2014).

Our previously validated rodent model (Bleier et al., 2013, Bleier et al., 2015) was designed to mimic human mucosal graft reconstruction. We first confirmed that the mucosal grafts were viable and well tolerated in all rats. We next used complementary Cy5 labeling and hybridization assays to confirm successful AntagoNAT delivery within critical end-target rat brain regions relevant to PD. Our BDNF ELISA data then verified that this delivery was effective resulting in BDNF protein upregulation in all regions of the brain. However, after statistical analysis, the increase in BDNF protein levels was statistically significant only in the substantia nigra and cerebellum region of the brain. This was due to the variability in measured BDNF protein levels from other parts of the brain (olfactory bulbs, striatum and hippocampus). Also, both substantia nigra and cerebellum demonstrated significant BDNF protein upregulation bilaterally in the AT-LIPO group whereas this increase in BDNF levels was unilateral for cerebellum in the AT-SALINE group. This can be attributed to better distribution of AT’s obtained with liposomal group as compared to AT-SALINE group. Though not statistically significant, BDNF AT liposomes did results in increased BDNF protein levels in all brain regions as compared to negative control group and BDNF AT in saline control group. Therefore, we decided to utilize BDNF AT liposomes for efficacy studies in 6-OHDA rat model of Parkinson’s disease. We applied the same delivery technique in the 6-OHDA model of PD. Using western blot analysis, we found that BDNF AT’s delivered using liposomes (AT-LIPO) were capable of preserving TH protein expression in both the striatum and substantia nigra confirming its therapeutic neuroprotective effect.

One unexpected finding was that saline outperformed the liposomal formulation in the hippocampus region ipsilateral to the mucosal graft. This finding may be due to differences in fiber tract orientation and density within the hippocampus which facilitated diffusion of the saline solution relative to the denser gray matter within the striatum and substantia nigra. A second interesting result was the lack of strong correlation between BDNF-AT distribution and subsequent BDNF protein upregulation. This is not entirely surprising as the AntagoNATs only act to derepress expression and thus rely on intrinsic regulatory pathways to promote transcription and translation which likely differ between brain sub regions (Modarresi et al., 2012; Rusk, 2012). This finding therefore serves to support the concept that AntagoNATs may be associated with less toxicity than recombinant protein delivery, as they will only act on cells programmed to express the protein of interest.

Our data confirm that AntagoNATs can be used to upregulate BDNF expression both *in vitro* and *in vivo* within key end target brain regions germane to PD. We have further shown that the common endonasal endoscopic skull base reconstructive technique of mucosal grafting can be adopted to overcome the inability of AntagoNATs to cross the BBB and that liposomal encapsulation enhances both BDNF AntagoNAT distribution and efficacy within the brain. Finally, our results demonstrate that liposome encapsulated BDNF AT’s are capable of exerting a neuroprotective effect in a rat 6-OHDA model of PD. More generally, given the ease of translation of these findings into clinical practice, our work suggests that transmucosal oligonucleotide delivery may provide a novel and much needed therapeutic option for patients suffering from both PD and other neurodegenerative diseases.

## Data Availability

All data needed to evaluate the conclusions in the paper are present in the paper and/or Supplementary Material. Additional data available from authors upon request.
