# Supplementary material for: Endonasal CNS Delivery System for Blood-Brain Barrier Impermeant Therapeutic Oligonucleotides Using Heterotopic Mucosal Engrafting
Source: Front Pharmacol. 2021 Apr 19;12:660841. doi: 10.3389/fphar.2021.660841 (PMC8090932; doi:10.3389/fphar.2021.660841)
Supplement: Supplementary file 1 [file datasheet1.pdf]

Supplementary Material

Supplementary Figures

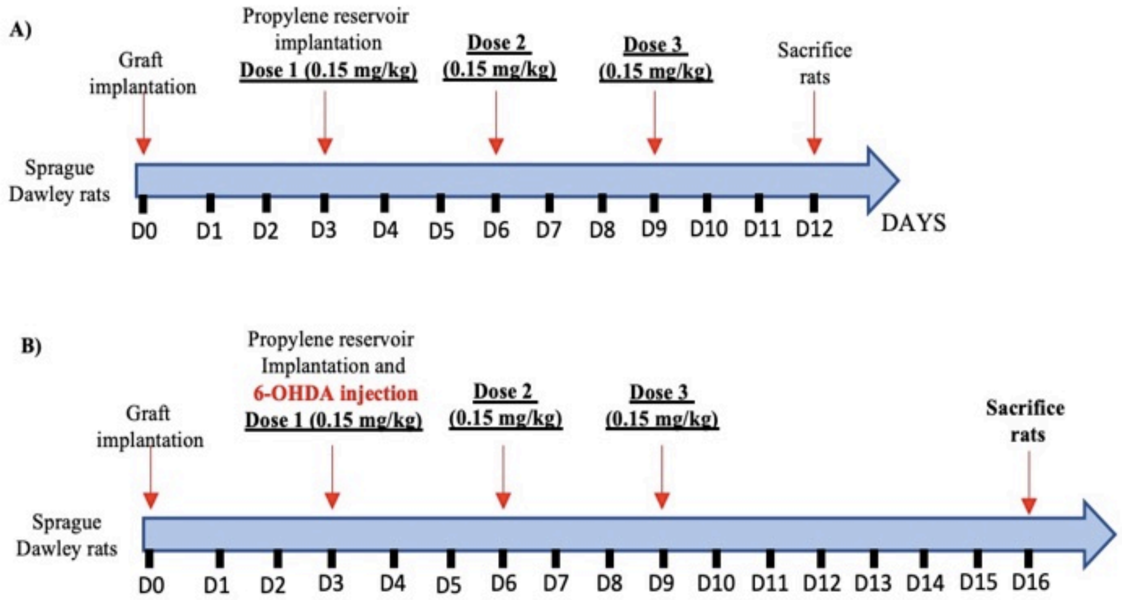

Fig S1: In vivo experimental timeline for A) Trans-nasal mucosal rat graft model and B) rat 6-hydroxydopamine (6-OHDA) heterotopic mucosal graft model of Parkinson's disease. Rats were dosed with 0.15 mg/kg of either BDNF AT saline or liposomal formulations.

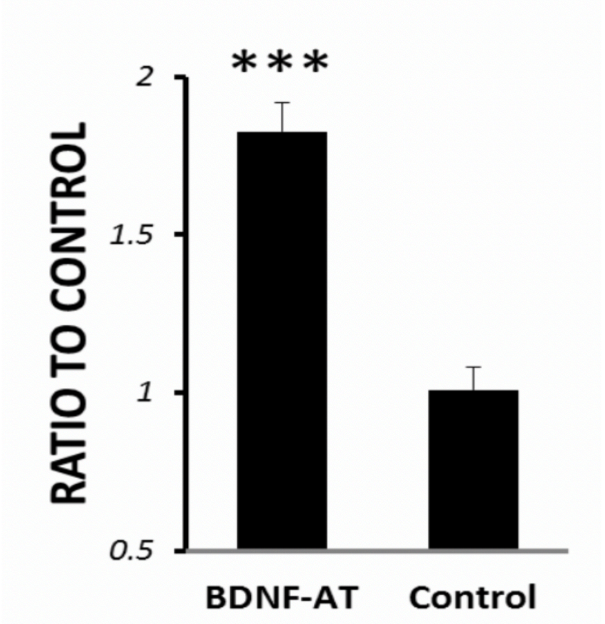

Fig S2: Upregulation of BDNF in rat schwannoma cell line RT4-D6P2T after treatment with 20 nM of BDNF-AT compared to inactive control oligonucleotide (real time PCR data, n=6, *** - t-test p<0.001).

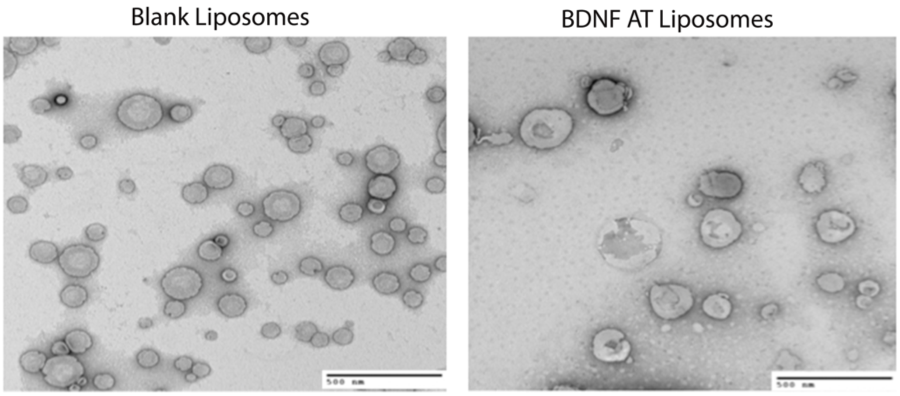

Sample	Average size (nm)	Average Polydispersity index	Average charge (mV)	% Encapsulation efficiency
Blank cationic Liposomes	206.5	0.208	33.4	NA
BDNF AT cationic liposomes	229.44 ± 17.6	0.235 ± 0.04	37.28 ± 3.3	~100%

Fig S3: TEM (uranyl acetate) images and characterization data (average size, PDI and charge) for the blank (no BDNF AT) (n=3) and BDNF-AT Liposomes (n=3).

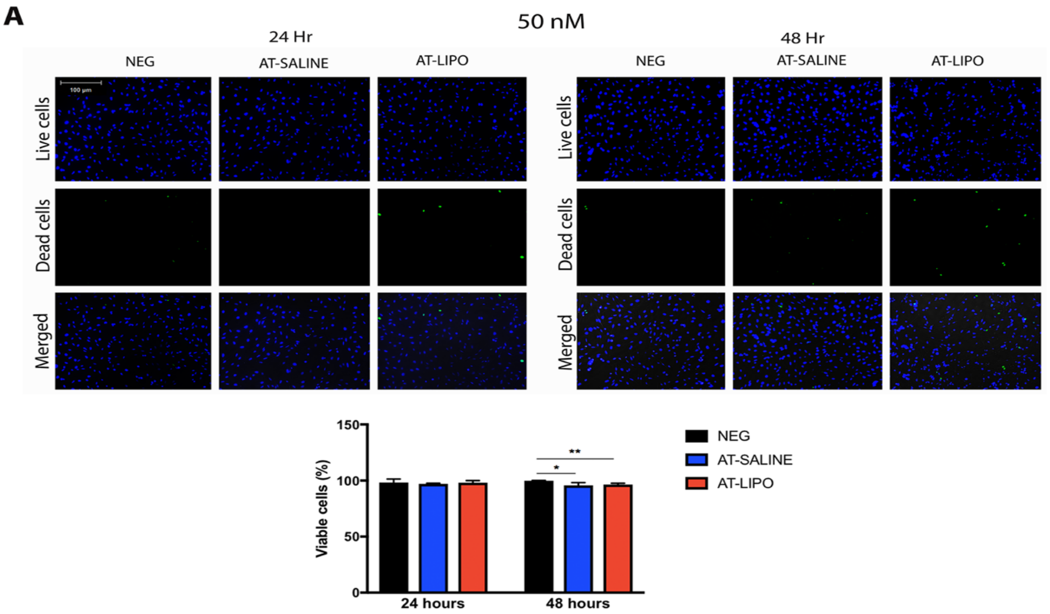

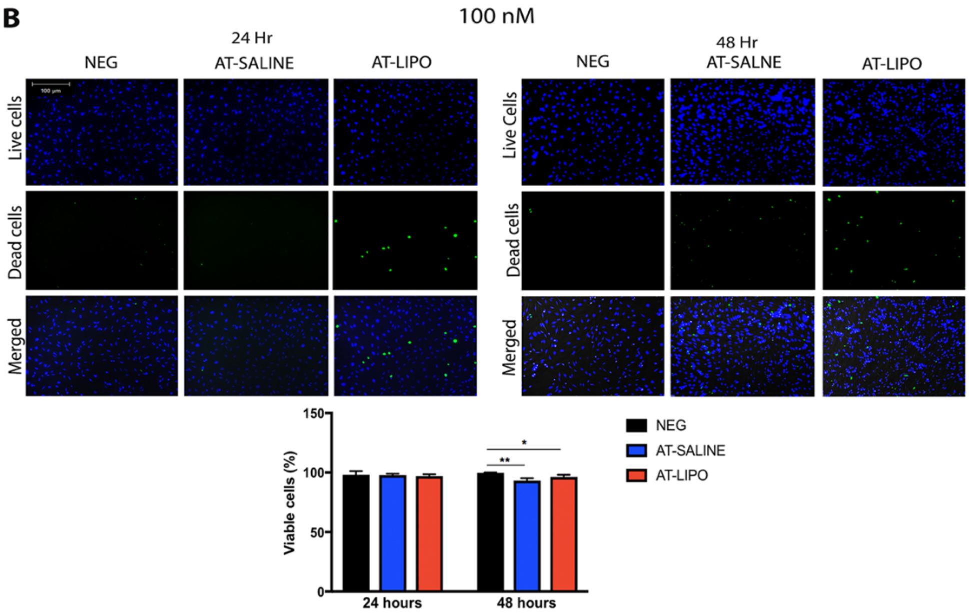

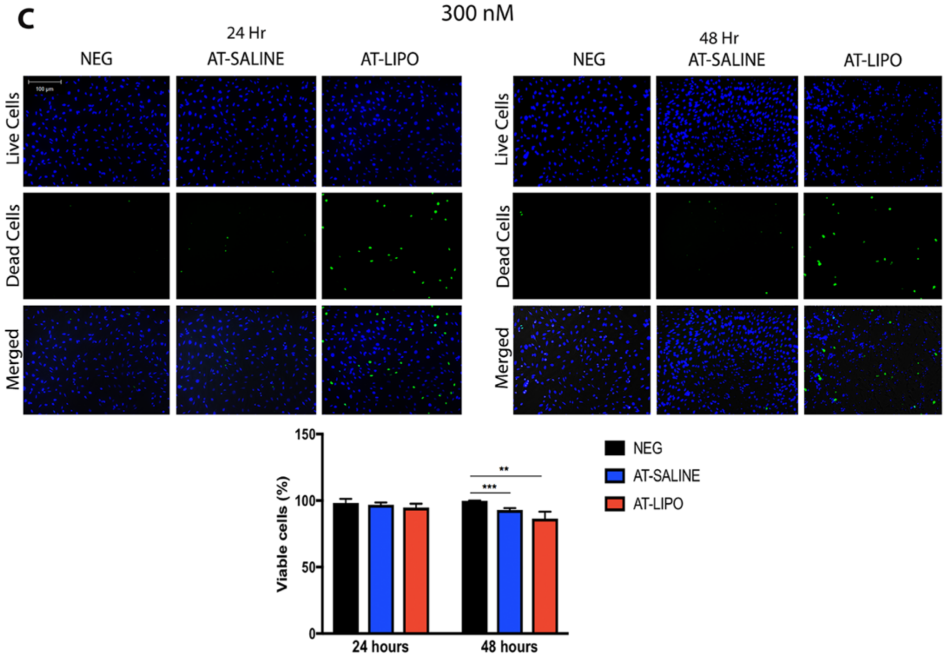

Fig S4: A-C. Live/dead assay in RT4-D6P2T rat schwannoma cells after exposure to 50-300nM of vehicle control (NEG), liposome encapsulated BDNF-AT (AT-LIPO), and BDNF-AT in saline (AT-SALINE) for 24-48 hours demonstrating greater than 80% viability at the doses and time-points studied. (Data presented as mean ± SD, Student's t-test).

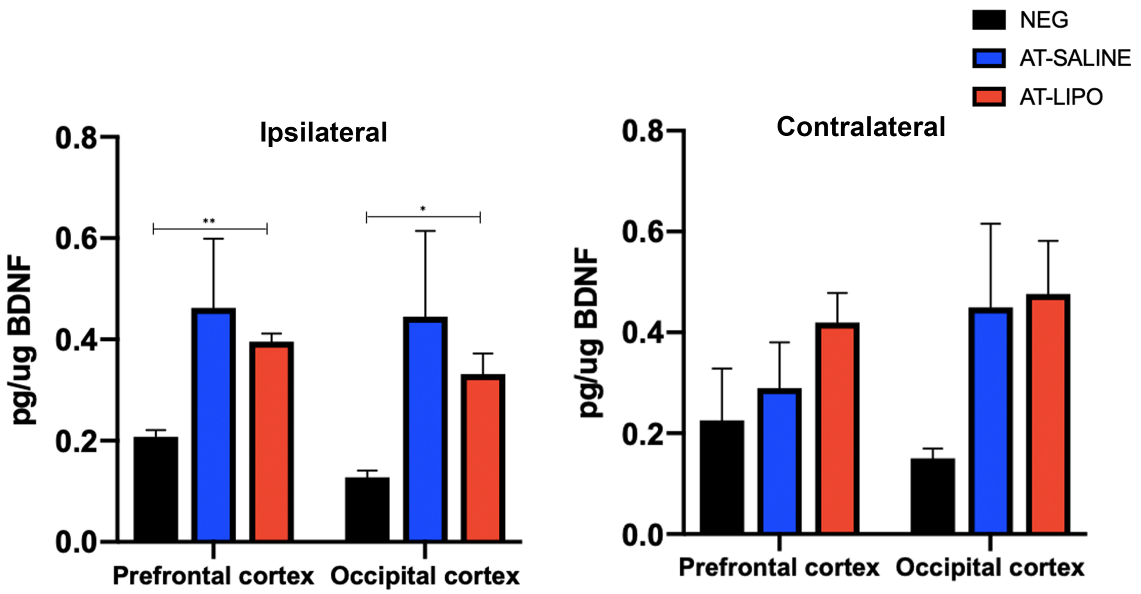

Fig S5. Bar graphs quantifying BDNF protein upregulation in ipsilateral and contralateral cortical regions of rat brain among the liposomal (AT-LIPO, n=4) and saline (AT-SALINE, n=4) groups as compared to negative control (NEG, * $p<0.05$, ** $p<0.01$; two-tailed unpaired Student's t-test). Data are presented as mean ± SD.
